# Assessing longitudinal housing status using Electronic Health Record data: a comparison of natural language processing, structured data, and patient-reported history

**DOI:** 10.3389/frai.2023.1187501

**Published:** 2023-05-24

**Authors:** Alec B. Chapman, Kristina Cordasco, Stephanie Chassman, Talia Panadero, Dylan Agans, Nicholas Jackson, Kimberly Clair, Richard Nelson, Ann Elizabeth Montgomery, Jack Tsai, Erin Finley, Sonya Gabrielian

**Affiliations:** ^1^Informatics, Decision-Enhancement and Analytic Sciences (IDEAS) Center, Salt Lake City Veterans Affairs Healthcare System, Salt Lake City, UT, United States; ^2^Division of Epidemiology, University of Utah, School of Medicine, Salt Lake City, UT, United States; ^3^Center for the Study of Healthcare Innovation, Implementation and Policy (CSHIIP), Greater Los Angeles Veterans Affairs Healthcare System, Los Angeles, CA, United States; ^4^Department of Medicine, David Geffen School of Medicine, University of California, Los Angeles, Los Angeles, CA, United States; ^5^Desert Pacific Mental Illness Research, Education, and Clinical Center (MIRECC), Veterans Affairs Greater Los Angeles, Los Angeles, CA, United States; ^6^Department of Epidemiology, Fielding School of Public Health, University of California, Los Angeles, Los Angeles, CA, United States; ^7^Department of Community Health Sciences, Fielding School of Public Health, University of California, Los Angeles, Los Angeles, CA, United States; ^8^Department of Medicine Statistics Core, David Geffen School of Medicine, University of California, Los Angeles, Los Angeles, CA, United States; ^9^United States Department of Veteran Affairs, Birmingham Veterans Affairs Health Care System, Birmingham, AL, United States; ^10^School of Public Health, University of Alabama at Birmingham, Birmingham, AL, United States; ^11^National Homeless Programs Office, United States Department of Veterans Affairs, Washington, DC, United States; ^12^Department of Psychiatry and Biobehavioral Sciences, David Geffen School of Medicine, University of California, Los Angeles, Los Angeles, CA, United States

**Keywords:** homelessness, electronic health records, natural language processing, veterans affairs, social determinants of health

## Abstract

**Introduction:**

Measuring long-term housing outcomes is important for evaluating the impacts of services for individuals with homeless experience. However, assessing long-term housing status using traditional methods is challenging. The Veterans Affairs (VA) Electronic Health Record (EHR) provides detailed data for a large population of patients with homeless experiences and contains several indicators of housing instability, including structured data elements (e.g., diagnosis codes) and free-text clinical narratives. However, the validity of each of these data elements for measuring housing stability over time is not well-studied.

**Methods:**

We compared VA EHR indicators of housing instability, including information extracted from clinical notes using natural language processing (NLP), with patient-reported housing outcomes in a cohort of homeless-experienced Veterans.

**Results:**

NLP achieved higher sensitivity and specificity than standard diagnosis codes for detecting episodes of unstable housing. Other structured data elements in the VA EHR showed promising performance, particularly when combined with NLP.

**Discussion:**

Evaluation efforts and research studies assessing longitudinal housing outcomes should incorporate multiple data sources of documentation to achieve optimal performance.

## 1. Introduction

Social determinants of health (SDoH) significantly impact patients' health and quality of life. Housing status is a key SDoH and ending homelessness among United States Veterans is a national priority for the Department of Veterans Affairs (VA), which provides a breadth of health and housing services for homeless-experienced Veterans (HEVs). To evaluate the effectiveness of VA homeless services, assessing short- and long-term housing outcomes is essential. However, to date, most studies that assess housing outcomes require collecting repeated patient-reported measures of housing status, which are costly and challenging to obtain.

In VA and other integrated healthcare systems, the Electronic Health Record (EHR) is a potentially valuable source of data regarding longitudinal housing outcomes. However, using EHR data for this purpose is challenging due to measurement error, missing data, and other complexities (Botsis et al., 2010; Wells et al., 2013; Glicksberg et al., 2018) which can bias outcomes assessed using longitudinal analyses (Lin et al., 2004; Pullenayegum and Lim, 2016; Lokku et al., 2021). SDoH are often recorded in the EHR using free-text clinical narratives (Organization, 2004; Gundlapalli et al., 2013, 2015; Peterson and Gundlapalli, 2015; Conway et al., 2019; Chapman et al., 2021; Hatef et al., 2022; Lybarger and Yetisgen, 2023; Tsai et al., 2022), and several studies have developed methods for extracting housing data from clinical texts (Gundlapalli et al., 2013; Conway et al., 2019; Chapman et al., 2021; Hatef et al., 2022; Lybarger and Yetisgen, 2023). In VA, one such system is Relative Housing Stability in Electronic Documentation (ReHouSED) (Chapman et al., 2021), a Natural Language Processing (NLP) system developed to extract housing stability from the EHR to evaluate VA's homelessness prevention and rapid rehousing program. ReHouSED demonstrated higher validity for identifying homeless status compared to International Classification of Diseases 10^th^ Edition (ICD-10) codes, a set of standardized codes representing clinical diagnoses and symptoms published by the World Health Organization (Organization, 2004).

However, there are several challenges in applying ReHouSED to study housing outcomes. First, the system may need to be adjusted for particular patient cohorts or evaluating specific services. It was originally designed for HEVs engaged in a rapid rehousing program; HEVs enrolled in other homeless services may have different EHR note structures or linguistic patterns. Second, missing data may cause bias when using ReHouSED for measuring outcomes. Information is only recorded in the EHR when patients present for care, which may occur more frequently for some patients than others. This produces observations at highly irregular intervals rather than the fixed, regularly spaced assessments that are ideal for longitudinal data collection, which can lead to biased analyses unless methods account for missing data (Pullenayegum and Lim, 2016; Lokku et al., 2021). Third, measurement error is ubiquitous in studies that use EHR data, particularly when using NLP to extract information from complex free text. While NLP is often designed to improve upon the shortcomings of structured data, misclassification is still present. This is especially true for complex variables such as longitudinal housing outcomes.

In the rapid rehousing context, ReHouSED achieved moderate accuracy (average positive predictive value and sensitivity of 65.3 and 68.1, respectively) and expert annotators achieved modest inter-annotator agreement (Cohen's Kappa = 0.7) (Chapman et al., 2021), demonstrating the complexity of the task. The accuracy of housing status classification can potentially be improved by combining NLP classifications with other EHR variables (e.g., ICD-10 codes) (Gundlapalli et al., 2015; Peterson and Gundlapalli, 2015; Wang et al., 2016; Nelson et al., 2018; Tsai et al., 2022). However, the accuracy of these data elements, as well as the best combination of indices, is not well-studied, in part due to the challenge of constructing a reference standard.

We aimed to develop a “best practice” for assessing longitudinal housing instability using observational EHR data as part of a quality improvement initiative targeting VA's Grant and Per Diem (GPD) case management aftercare program (hereafter, “Aftercare”). In this program, VA partners with community-based homeless service agencies to provide 6 months of case management for HEVs undergoing housing transitions (e.g., from institutional settings to independent housing). For a cohort of Aftercare patients in Southern California, we collected patient-reported housing history for a 2-year period. We then extracted six indicators of housing instability from the VA EHR: clinical note classifications of housing status obtained using ReHouSED tailored for this cohort (Chapman et al., 2021); ICD-10 codes for homelessness; notations of homeless service use found in outpatient visits; inpatient admissions associated with homelessness (e.g., residential treatment programs); a universal screening tool to assess housing instability; and data from VA's homeless registry. We compare the validity of each indicator of housing instability, considering the patient-reported data as a gold standard, and discuss implications for evaluations of housing interventions.

## 2. Materials and methods

### 2.1. Setting and ethics

Our cohort consisted of 386 VA Greater Los Angeles patients who engaged in Aftercare between 10/1/2019 and 1/4/2021. This cohort was enrolled in a parent project evaluating the implementation of Critical Time Intervention, an evidence-based, structured, and time-limited case management practice (Herman et al., 2000; Gabrielian et al., 2022). All project activities were reviewed by VA's Central Institutional Review Board and designated as quality improvement.

We extracted patient demographics for the entire cohort including age, race, and ethnicity from administrative data collected as part of Aftercare. Additionally, we identified recent diagnoses of psychiatric and substance use disorders using ICD-10 diagnosis codes derived from the VA's National Psychosis Registry (Blow et al., 2004). We included the following conditions in our analysis: alcohol use disorders; drug use disorders; schizophrenia spectrum and other psychotic disorders; bipolar disorders; major depressive disorder; anxiety disorders; and post-traumatic stress disorders. The complete list of ICD-10 codes can be found in the Supplementary material. Of note, we did not assess for the presence of dementia or other major neurocognitive disorders (exclusion criteria from the housing program in which this cohort was engaged); mental retardation (which is incompatible with military service); or personality disorders (which are inaccurately captured in VA administrative data). Diagnosis codes were retrieved from outpatient and inpatient settings in the year preceding the patient lookback period (defined in the following paragraph).

### 2.2. Patient-reported housing outcomes

We recruited a random subsample of 61 patients from the cohort for detailed telephone assessments of their housing status from 7/1/2020 and 6/30/2022. The goal was to create a reference standard to enable refinement of EHR methodologies for assessing housing status. We sent recruitment letters to 188 randomly selected Veterans, 19 of whom opted into the study. 158 of the remaining Veterans received follow-up recruitment calls and 41 volunteered to participate.

Following verbal informed consent, assessments were conducted with the Residential Time-Line Follow Back (TLFB) inventory, a validated instrument that collects retrospective housing status (Mendelson et al., 2010). The TLFB assigns codes for 34 different housing types (e.g., “On the street or in other outdoor place,” “Own apartment or house”) and classifies each type to one of four categories: “Literal Homelessness,” “Temporary,” “Stable,” and “Institutional.” In these analyses, we collapsed “Literal Homelessness” and “Temporary Housing” into a single “Unstable” category. Except for inpatient admissions coded as “Hospital (medical only),” any “Institutional” code was also considered to be “Unstable.” This meant that short-term institutional facilities, such as residential programs or crisis housing, were considered unstable.

Using standardized TLFB procedures, participants reported all changes in housing status over the specified period. We defined patient episodes as a continuous period spent “stable” or “unstable.” For example, patients who were stably housed during the entire period had a single episode (even if they changed addresses or moved to a different subcategory of stable housing), whereas a patient who was stably housed at the beginning of the period but then became unstably housed for the rest of the period had two episodes.

### 2.3. EHR indicators of housing status

EHR data for all patients in this subsample was obtained from VA's Corporate Data Warehouse (CDW), a national repository of demographics, diagnoses, clinical narratives, and other clinical and administrative data. Additional data was retrieved from the VA's homeless service registry and linked to CDW data.

#### 2.3.1. NLP system

**Figure 1:**
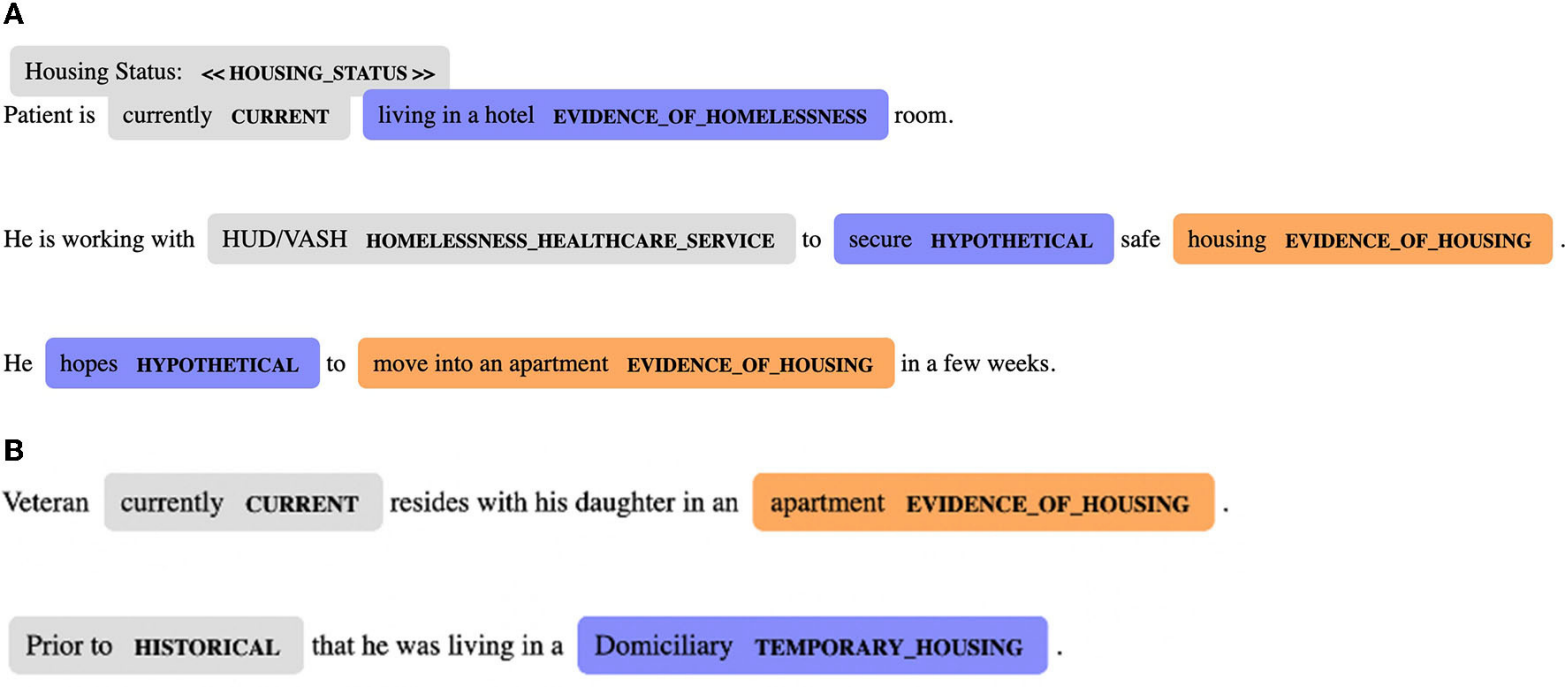
**(A)** A clinical note classified by ReHouSED as “Unstable.” The note states that the patient is living in a hotel and hopes to move into stable housing soon. HUD-VASH, HUD-Veterans Affairs Supportive Housing. **(B)** A clinical note classified by ReHouSED as “Stable.” The note mentions the patient's history of living in unstable housing but states that the patient is currently stably housed.

We used ReHouSED to extract housing status from clinical notes in the subset of patients who completed telephone interviews (Chapman et al., 2021). ReHouSED is a rule-based system implemented in medspaCy (Eyre et al., 2021) that was originally developed to extract housing outcomes from HEVs participating in VA's rapid rehousing program. Rules are hand-crafted to define semantic phrase and syntactic patterns, matching entities related to homelessness (e.g., “sleeps in the park,” “needs shelter”) and housing stability (e.g., “lives in an apartment,” “no concerns about housing“). Each entity is then linked to any linguistic modifiers such as phrases indicating negation (e.g., “not currently”) or risk (e.g., “worried about being evicted”). Notes are also parsed to identify the clinical note sections, such as past medical history or social history. This contextual information is used to interpret whether each entity is referring to the patient's current housing status and whether they are stably housed. Based on text in a note, each note is assigned one of three housing status classifications: “Stable,” “Unstable,” or “Unknown.” The last of these classifications refers to notes that include some mention of housing or discussion of a patient's history of housing instability but have no discernible statement of the patient's current housing status. Examples of documents classified as “Unstable” and “Stable,” respectively, are shown in Figure 1.

Using a random sample of 250 notes from the larger cohort (*n* = 386), we tailored ReHouSED to fit housing outcome classifications pertinent to Aftercare. First, we identified clinical note templates and phrases related to the receipt of VA permanent supportive housing services (independent housing with financial subsidies and supportive services). Though ReHouSED initially classified permanent supportive housing as “Unstable,” we conceptualized permanent supportive housing as a positive (“Stable”) outcome for Aftercare patients. Second, while ReHouSED prioritized mentions of stable housing over mentions of homelessness or temporary housing in a clinical note, we modified the document classification logic to prioritize current mentions of VA's residential treatment program for HEVs (known as the Domiciliary); for HEVs engaged in Aftercare, enrollment in residential treatment was considered a negative (“Unstable”) outcome. Last, based on a review of this sample of notes, we added a small number of additional concepts that were not included in the original ReHouSED system (e.g., “currently incarcerated,” “sober home”).

We processed all notes mentioning housing keywords for interviewed patients during the 2-year assessment period. The housing keywords and additional exclusion criteria are the same as those described by Chapman et al. (2021). If multiple notes mentioning housing were present on a single day, we classified the encounter as “Unstable” if at least half of the notes were classified as “Unstable” after excluding “Unknown” notes. If fewer than half were classified as “Unstable,” or if there were no notes classified as “Stable” or “Unstable,” the housing status that day was deemed “Stable.”

#### 2.3.2. Structured EHR data

We abstracted demographic data (age, gender, race, ethnicity) from the EHR. We also obtained structured EHR data elements that indicate housing instability: ICD-10 codes for behavioral health disorders (psychiatric diagnoses and substance use disorders); outpatient administrative data that indicate receipt of homeless services; inpatient administrative data that describe admission to programs for HEVs; and a homelessness screening tool. Each data element is detailed below. Specific value sets for each data element are provided in the Supplemental material.

*ICD-10 codes:* Several ICD-10 codes associated with outpatient visits or inpatient care indicate homelessness or risk of homelessness (e.g., “Z59.0: Homelessness, unspecified”). We retrieved all ICD-10 codes pertaining to homelessness or risk of homelessness during the study period. We conceptualized a patient as unstable if there was a homeless-associated ICD-10 code on a given day.

Outpatient administrative data: In VA EHR, the type of outpatient clinical service is coded. We identified codes indicating use of VA homeless services and considered an encounter unstable if the Veteran received care from any of these services.

Inpatient administrative: For all hospital stays in the study cohort, we identified residential treatment programs for HEVs (conceptualized as inpatient admissions in VA, e.g., the “Domiciliary Care for Homeless Veterans (DCHV) program”).

Homelessness screener: The Homelessness Screening Clinical Reminder (HSCR) is an instrument delivered to all Veteran outpatients to routinely screen for recent housing instability or risk of housing instability (Montgomery et al., 2022). Responses to this screener are saved in the EHR as structured data elements. We identified positive responses from interviewed Veterans.

#### 2.3.3. Homeless service registry data

The VA maintains an administrative database of homeless services provided to Veterans by the VA or its community partners, referred to as the homeless service registry (HOMES). We queried this database for enrollment and exit dates into housing assistance programs and considered patients to be unstably housed during their enrollment period.

### 2.4. Analyses

#### 2.4.1. TLFB data

Using TLFB data, we calculated the count, percent of episodes, and total person-days spent in each of three categories: unstable, stable, and institutional. Because days spent in institutional settings (e.g., hospital admissions not related directly to homelessness) were expected to be uncommon and captured using inpatient administrative data, episodes assigned to this category were excluded from further analyses. We also derived a binary variable indicator whether the patient reported housing instability at any point in the 2-year assessment period. We measured the association between housing instability at any point with baseline characteristics (i.e., demographic variables and psychiatric diagnoses) using a logistic regression model.

#### 2.4.2. VA service use frequency and type

Analyses using EHR data depend on documentation of patients' service use, leading to missing data on days when patients are not engaged with the VA health system. To assess patterns of service utilization and corresponding rates of missingness, we calculated descriptive statistics of the frequency of encounters, defined as any inpatient or outpatient service documented in the EHR. We calculated the count and proportion of patients, person-days, and person-months with at least one encounter in VA during the data collection period. We also calculated the mean and standard deviation of the number of encounters per month. To assess the number of clinical notes discussing housing, we repeated each calculation limited to encounters that contained notes classified by ReHouSED as “Stable” or “Unstable.” To explore whether rates of encounter frequency differed between stably and unstably housed individuals, which could cause bias in longitudinal analyses, we stratified these statistics by whether they were ever unstably housed during the data collection period. We visually characterized encounter frequency in these two groups by plotting encounters over time using an abacus plot (Lokku et al., 2021).

#### 2.4.3. Validity of EHR indicators

We assessed the accuracy of each individual EHR indicator for differentiating stable vs. unstable housing. First, we calculated the proportion of ever unstably housed and never unstably housed patients who had each indicator. Indicators found to be present for less than two unstably housed patients were excluded from subsequent analyses. For the remaining indicators, we calculated encounter- and month-level sensitivity and specificity for each indicator. For encounter-level performance, we calculated sensitivity as the proportion of encounters during an episode of unstable housing where that indicator was present, and specificity as the proportion of encounters during stable episodes that did not have the indicator. We considered each of the EHR indicators individually as well as different combinations of EHR indicators (e.g., NLP and ICD-10 codes denoting housing instability). Bootstrapping was used to construct 90% confidence intervals.

A limitation of measuring the performance of EHR indicators at the encounter level is that many VA visits may not include documentation of a patient's housing status. For example, visits for medical/surgical procedures generally do not include documentation of housing status and would be counted as false negatives in the encounter-level sensitivity. To account for this, we first limited the data to encounters where the patient had at least one note classified as “Stable” or “Unstable” by ReHouSED; this required an explicit NLP classification of housing status and does not equate the absence of documented unstable housing to stable housing. Second, we aggregated data to patient-months. For each patient, the patient's housing status was considered unstable if he/she reported an episode of unstable housing that overlapped with that month. A patient-month was classified as unstable if at least half of a patient's encounters during that time had indicators of instability. This month-level analysis was limited to patient-months that had at least one VA service use.

Unlike EHR data, the HOMES data records start and end dates of service use, removing the need for a patient to present for medical care to ascertain their housing status. To compare HOMES vs. EHR data, we restricted HOMES records to days in which patients had an EHR-recorded encounter, but separately calculated the total proportion of person-days (with or without an encounter) captured using HOMES data.

## 3. Results

Table 1 summarizes demographics for patients who provided self-reported housing history (“interviewed”) vs. those who did not. Among interviewed patients, most (63.9%) were >60 years old and 85.2% were male. Over half (54.1%) were African American. Among the entire cohort, the most common psychiatric diagnoses were major depressive (27.2%) and post-traumatic stress disorders (24.9%), with a smaller proportion of patients demonstrating evidence of drug use (13.2%), alcohol use (16.1%), or psychotic spectrum disorders (4.9%).

**Table 1:** Sample demographics.

**Characteristic**	**Interviewed**		
	**Yes, *n* = 61**	**No, *n* = 325**	**Overall, *n* = 386**
Age (mean, SD, in years)	60.6, 11.3	59.7, 14.7	59.8, 14.2
< 40 years (*n*, %)	2 (3.3%)	45 (13.8%)	47 (12.2%)
40–50 years (*n*, %)	11 (18.0%)	39 (12.0%)	50 (13.0%)
50–60 years (*n*, %)	9 (14.8%)	43 (13.2%)	52 (13.5%)
>60 years (*n*, %)	39 (63.9%)	198 (60.9%)	237 (61.4%)
**Self-identified gender (n, %)**			
Female	9 (14.8%)	28 (8.6%)	37 (9.6%)
Male	52 (85.2%)	297 (91.4%)	349 (90.4%)
**Race (** ***n*** **, %)**			
American Indian/Alaska Native	0 (0.0%)	11 (3.4%)	11 (2.8%)
Black/African American	33 (54.1%)	135 (41.5%)	168 (43.5%)
Native Hawaiian/Other Pacific Islander	1 (1.6%)	3 (0.9%)	4 (1.0%)
White	24 (39.3%)	147 (45.2%)	171 (44.3%)
Missing/Other	3 (4.9%)	29 (8.9%)	32 (8.3%)
**Ethnicity (** ***n*** **, %)**			
Hispanic or Latino	3 (4.9%)	40 (12.3%)	43 (11.1%)
Not Hispanic or Latino	56 (91.8%)	269 (82.8%)	325 (84.2%)
Missing/Other	2 (3.3%)	16 (4.9%)	18 (4.7%)
**Psychiatric and substance use disorders (** ***n*** **, %)**			
Bipolar disorder	1 (1.6%)	8 (2.5%)	9 (2.3%)
Major depressive disorder	19 (31.1%)	86 (26.5%)	105 (27.2%)
Anxiety disorder	9 (14.8%)	56 (17.2%)	65 (16.8%)
Post-traumatic stress disorders	16 (26.2%)	80 (24.6%)	96 (24.9%)
Schizophrenia spectrum and other psychiatric disorders	2 (3.3%)	17 (5.2%)	19 (4.9%)
Alcohol use disorder	8 (13.1%)	54 (16.6%)	62 (16.1%)
Drug use disorder	9 (14.8%)	42 (12.9%)	51 (13.2%)

N, number of patients; SD, standard deviation.

### 3.1. Patient-reported housing status

Table 2 summarizes patient-reported housing episodes, stratified as institutional, unstable, or stable. Most of the cohort was stably housed during the period examined, with most patients (*n* = 56, 91.8% of all patients) reporting stable housing at least once during the period, for a total of 35,953 person-days. Fewer (*n* = 12, 19.7%) patients reported being unstably housed at least once, for a sum of 3,803 person-days. Episodes of stable housing typically lasted longer than episodes of unstable housing (mean 486 days vs. 200 days). Very few (*n* = 4, 6.6%) patients reported time spent in institutions, accounting for a total of 112 person-days. These 112 days (presumed to be hospitalizations) are excluded in subsequent analyses.

**Table 2:** Summary of patient-reported housing status episodes from 7/1/2020 - 6/30/2022, obtained using the Residential Time-Line Follow Back (TLFB) inventory on a cohort of 61 patients.

**Characteristic**	**Institutional**	**Stable**	**Unstable**
Number (%) of patients reporting at least one housing episode (total number of patients = 61)	4 (6.6%)	56 (91.8%)	12 (19.7%)
Number (%) of episodes in each category (total number of episodes = 97)	4 (4.1%)	74 (76.3%)	19 (19.6%)
Number (%) person days spent in each category (total number of person-days = 39,868)	112 (0.3%)	35,953 (90.2%)	3,803 (9.5%)
**Episode duration, in days**			
Minimum	1	38	3
Maximum	74	729	667
Mean (SD)	28 (33.1)	485.9 (253.4)	200.2 (226.6)
Median	18	578	91

SD, Standard deviation.

The coefficients for the logistic regression model of housing instability at any point are shown in Table 3. There was no significant association between housing instability and any demographic variables (i.e., race, ethnicity, age, or gender) and housing instability, but there was some evidence of higher odds of housing instability for patients diagnosed with one or more psychiatric disorders (odds ratio = 7.85, 90% confidence interval = [1.61, 56.4]), as well as one or more substance use disorders (22.7 [4.75, 146]).

**Table 3:** Coefficients for logistic regression model relating baseline characteristics and diagnoses and reporting housing instability at any point between 7/1/2020-6/30/2022 for a sample cohort 61 patients.

**Characteristic**	**OR**	**90% CI**
Age	0.99	0.93, 1.06
Ethnicity not Hispanic or Latino	REF	REF
Hispanic or Latino	0.10	0.00, 1.54
Race White	REF	REF
Non-white	0.30	0.06, 1.32
Gender Male	REF	REF
Female	1.01	0.08, 7.49
Any psychiatric disorder^*^	7.85	1.61, 56.4
Substance use disorder^**^	22.7	4.75, 146

OR, Odds ratio; CI, Confidence interval.

* Psychiatric disorders include bipolar disorder, major depressive disorder, anxiety disorders, post-traumatic stress disorders, or schizophrenia spectrum/other psychotic disorders.

** Substance use disorders include alcohol use disorder, cannabis use disorder, cocaine use disorder, opioid use disorder, hallucinogen use disorder, sedative use disorder, and other stimulants/psychoactives use disorders.

### 3.2. EHR encounters

**Figure 2:**
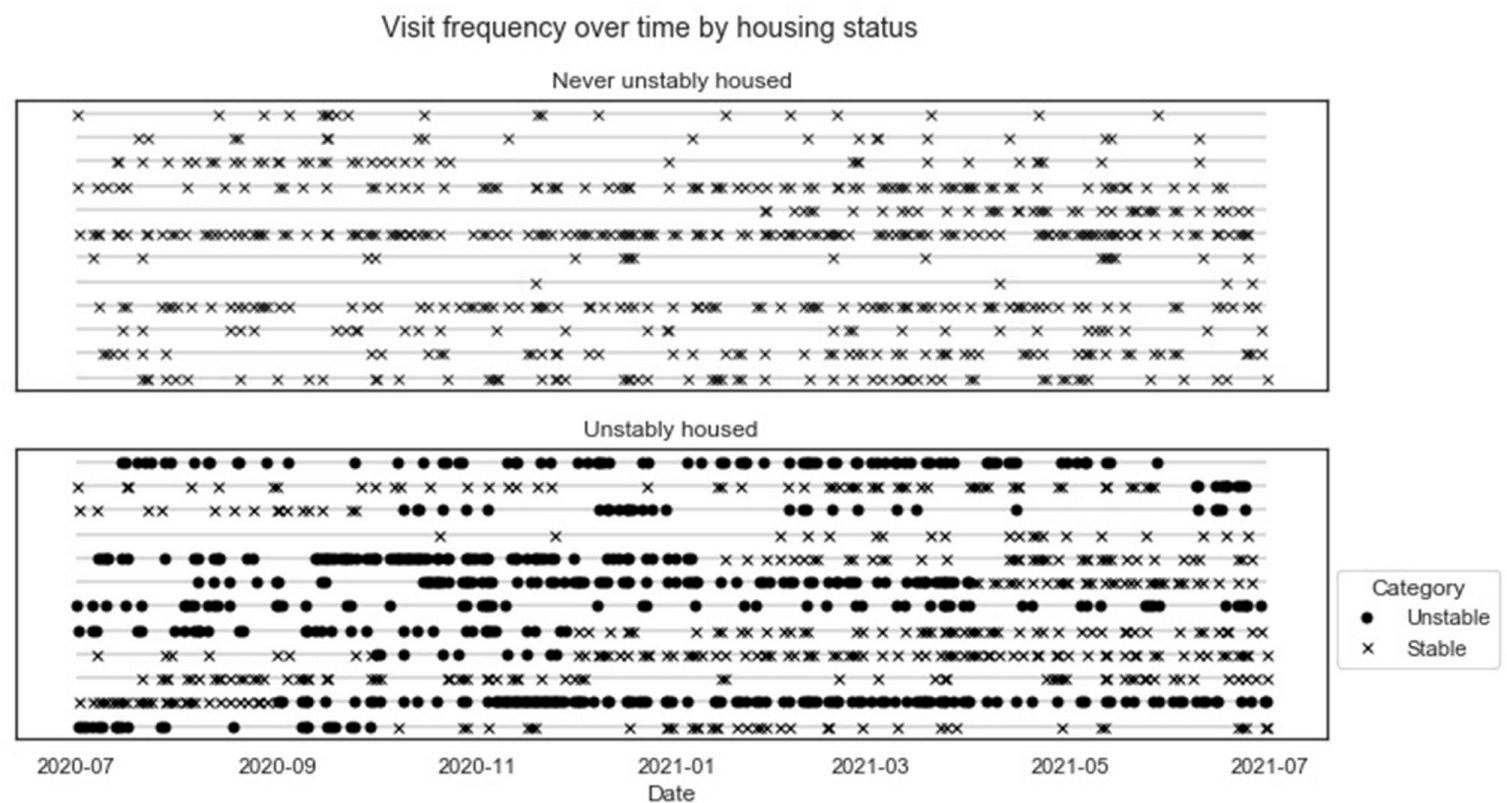
An abacus plot displaying the frequency of patient visits over time. The **top** panel displays visits for a subsample of patients who were stably housed during the first year of the assessment period. The **bottom** panel display panels for patients who were unstably housed at some point in the assessment period, with encounters on unstably housed days shown in solid circles.

Most (58) patients had an encounter at some point over the 2 years. Patients who experienced unstable housing had more encounters per month compared to patients who remained stably housed (mean 7.0 vs. 5.1, ratio = 1.37). Limiting to encounters with notes mentioning housing, this ratio increased slightly (mean 3.6 vs. 2.4, ratio = 1.5). Similarly, patients with unstable housing experiences had a higher probability of having at least one encounter in a given month. This difference in visit frequency is shown visually in Figure 2, which plots visit frequency over the 1st year of the study period for a randomly selected subsample of 12 patients with no unstable housing (top panel) and the 12 patients who reported unstable housing (bottom). Points represent an encounter at the specified time point, with shape representing the patient's reported housing status at the time (unstable encounters are marked by solid circles, while stable encounters are marked by an “x”). There is clear variation across patients in visit frequency. Unstable episodes are characterized by dense clusters of visits, while periods of stable housing tend to be sparser and more spread out, suggesting that this population of patients may interact with the VA healthcare system less frequently during long periods of housing stability

### 3.3. Validity of EHR indicators

Of the 12 patients who reported at least one unstably housed experience on the TLFB, 11 (91.6%) had some documentation of unstable housing over the assessment period, while 1 (8.4%) did not have any data elements indicating housing instability. NLP, ICD-10 codes, and outpatient administrative data were each present for all of these 11 patients, while the inpatient variables and the homelessness screener were each used with only 1 patient. Most (8/12, 66.7%) patients with unstable housing experiences were recorded in HOMES as having received homeless services.

We examined encounter- and month-level sensitivity and specificity for NLP, ICD-10 codes, outpatient data, and HOMES, as well as combinations of the structured EHR data and NLP. Table 4 shows the results for individual indicators, combinations of NLP and ICD-10 codes, and combinations of NLP and any structured data, including VA-specific data elements; data were restricted to person-days and months where the patient had a VA encounter. At the encounter level, NLP displayed higher sensitivity (0.197, bootstrapped 90% CI = [0.143, 0.251]) than ICD-10 codes (0.098 [0.039, 0.157]) and outpatient data (0.102 [0.074, 0.129]), but lower sensitivity than HOMES (0.268 [0.076, 0.459]). Bootstrapped confidence intervals for sensitivity were wide due to the small number of unstably housed patients The widest confidence intervals were observed for HOMES data due to high between-subject variation (i.e., only 66.7% of unstably housed patients were in the registry). Encounter-level specificity was highest for HOMES (0.969 [0.947, 0.990]) and outpatient data (0.967 [0.950, 0.983]), and lower for NLP (0.948 [0.936, 0.960]) and ICD-10 codes (0.943 [0.922, 0.964]).

**Table 4:** Sensitivity and specificity, and confidence intervals, for four EHR indicators of unstable housing at the encounter and month level.

	**Encounter**		**Encounters with notes documenting housing status**		**Month**	
**EHR indicator**	**Sensitivity (*****n*** = **796)**	**Specificity (*****n*** = **4,932)**	**Sensitivity (*****n*** = **228)**	**Specificity (*****n*** = **749)**	**Sensitivity (*****n*** = **114)**	**Specificity (*****n*** = **962)**
NLP	0.197 (0.143, 0.251)	0.948 (0.936, 0.960)	**0.689 (0.595, 0.782)**	0.658 (0.599, 0.717)	**0.421 (0.313, 0.529)**	0.876 (0.847, 0.906)
ICD-10	0.098 (0.039, 0.157)	0.943 (0.922, 0.964)	0.224 (0.095, 0.352)	0.862 (0.820, 0.904)	0.298 (0.207, 0.389)	0.858 (0.809, 0.907)
Outpatient admin	0.102 (0.074, 0.129)	0.967 (0.950, 0.983)	0.259 (0.190, 0.328)	0.919 (0.872, 0.965)	0.360 (0.238, 0.481)	0.878 (0.826, 0.931)
HOMES	**0.268 (0.076, 0.459)**	**0.969 (0.947, 0.990)**	0.311 (0.063, 0.560)	**0.928 (0.869, 0.986)**	0.254 (0.082, 0.427)	**0.964 (0.935, 0.992)**
NLP or ICD-10	**0.246 (0.187, 0.306)**	0.900 (0.873, 0.928)	**0.741 (0.653, 0.830)**	0.581 (0.523, 0.638)	**0.535 (0.433, 0.637)**	0.778 (0.727, 0.828)
NLP and ICD-10	0.049 (0.012, 0.086)	**0.991 (0.987, 0.995)**	0.171 (0.055, 0.287)	**0.940 (0.917, 0.963)**	0.184 (0.119, 0.250)	**0.956 (0.938, 0.974)**
Any structured	0.377 (0.206, 0.548)	0.897 (0.866, 0.927)	0.592 (0.410, 0.774)	0.768 (0.697, 0.838)	0.518 (0.416, 0.619)	0.775 (0.711, 0.840)
NLP or any structured	**0.450 (0.298, 0.602)**	0.861 (0.826, 0.895)	**0.846 (0.769, 0.924)**	0.531 (0.464, 0.599)	**0.640 (0.525, 0.756)**	0.712 (0.649, 0.775)
NLP and any structured	0.124 (0.068, 0.181)	**0.984 (0.978, 0.990)**	0.434 (0.272, 0.596)	**0.895 (0.853, 0.936)**	0.298 (0.218, 0.378)	**0.940 (0.916, 0.964)**

*n*, Number of encounters (columns 1-2), encounters with notes (columns 3-4), and patient-months (columns 5-6); NLP, Natural language processing; ICD-10, International Classification of Diseases (10th edition); HOMES, Homelessness service registry data.

^*^Structured data = ICD-10, outpatient data, or HOMES.

The highest individual and compositive sensitivity and specificity are shown in bold.

When limited to encounters with notes pertaining to housing, NLP had the highest sensitivity (0.689 [0.595, 0.782]) and the lowest specificity (0.658 [0.599, 0.717]). The other three indicators each saw increased sensitivity and decreased specificity, although the change was less extreme than for NLP. When aggregating to the month level, NLP again saw the highest sensitivity (0.421 [0.313, 0.529]). HOMES, which had the highest encounter-level sensitivity, had the lowest sensitivity at the patient-month level (0.254 [0.082, 0.427]) due the high percentage of patients (66.7%) who were not captured in this dataset. ICD-10 codes had the lowest sensitivity (0.298 [0.207, 0.389]) and lowest specificity (0.858 [0.809, 0.907]).

Composite measures using any of the three structured elements had higher sensitivity and lower specificity than any of the structured elements at each level of analysis. A similar pattern was observed when using NLP or any structured data. Requiring NLP and structured EHR data of housing instability achieved lower sensitivity and higher specificity than NLP or HOMES individually but maintained higher sensitivity as well as specificity than when using only ICD-10 codes and outpatient administrative data.

Across all person-days during the assessment period regardless of whether the patient had an encounter, HOMES had a sensitivity of 0.2 [0.067, 0.335] and specificity of 0.971 [0.954, 0.996]. The sensitivity achieved at the person-day level using the combination of all three EHR indicators (i.e., patients having at least one of NLP, ICD-10, or outpatient administrative data) was 0.06 [0.03, 0.08], showing an advantage of using HOMES administrative data that did not require patients to present for care.

## 4. Discussion

We compared patient-reported housing history with clinical and administrative data regarding housing status for a cohort of homeless-experienced VA patients. Our goal was to compare the validity of different data elements to identify best practices for assessing longitudinal housing outcomes using EHR data. Among the small number of patients who experienced housing instability in our cohort, most had EHR documentation of their housing status. Using NLP to supplement standard structured data elements with information recorded in clinical notes NLP led to more complete assessment of longitudinal housing outcomes. This is an important finding with methodologic implications for optimizing the validity of assessing patients' longitudinal housing outcomes using EHR data when patient-level data collection is not feasible due to sample size or resource constraints.

In these analyses, sensitivity and specificity varied by EHR extraction method. NLP generally had higher sensitivity than structured EHR data for capturing repeated occurrences of housing instability, but demonstrated lower specificity than some structured elements. ICD-10 codes, which are often used in epidemiologic studies, had lower sensitivity and specificity than most other indicators, including NLP. Combining NLP and ICD-10 codes increased sensitivity but decreased specificity. These findings build on prior work with ReHouSED in a distinct cohort of VA patients engaged in rapid rehousing (Chapman et al., 2021); at the patient-month level, both analyses provide evidence that ReHouSED performs better than ICD-10 codes in measuring housing instability.

The VA EHR contains data elements for documenting housing instability that are unique to VA. In particular, outpatient administrative data had higher specificity than NLP and higher sensitivity than ICD-10 codes. Combinations of these three elements could be used to tailor definitions to improve sensitivity or specificity as appropriate for a particular cohort or analysis. Additionally, while encounter- and month-level performance varied across different data elements, patient-level sensitivity was similarly high for NLP, ICD-10 codes, and outpatient administrative data, suggesting structured data may be sufficient for constructing coarse definitions of housing instability (e.g., identifying patients with a history of housing instability at any point in time).

When patients received services recorded in HOMES, those episodes of housing instability were captured with high sensitivity and specificity. However, this dataset does not capture an important segment of the population that is disengaged from VA homeless services; our data suggests that quality improvement leaders and researchers using HOMES to assess housing outcomes should consider complementing this data with EHR data elements. These findings parallel prior work (Tsai et al., 2022) comparing estimated prevalence of homelessness across VA, which found that utilizing multiple EHR data elements can improve ascertainment of housing instability.

When deciding how to define housing instability using EHR data, we suggest that specific analytic goals and the underlying prevalence of housing instability be taken into consideration. Analyses examining cohorts with low prevalence of housing instability, as we had here, may demand high specificity to avoid large numbers of false positives. Specificity can be improved by requiring multiple data elements to show evidence of housing instability or by favoring more specific data elements. When high sensitivity is more desirable, using NLP or the union of multiple data elements may be more effective. Attention should also be given to missing data, as EHR data depends on patients presenting for care. Patients experiencing housing instability may use care more frequently, leading to an imbalance in the degree of observation for stably and unstably housed patients. To avoid biased results, longitudinal analyses of housing instability using EHR data should consider utilizing methods for adjusting for missing data and irregular observations (Lin et al., 2004; Pullenayegum and Lim, 2016; Pullenayegum and Scharfstein, 2022).

This work has limitations. First, we performed these exploratory analyses on a small sample and thus our statistical analyses had had low power. Regardless, the detailed patient-reported housing history we obtained over a 2-year period in patients with homeless experiences is a valuable observational dataset and our findings will inform future work. As with any retrospective analyses using patient-reported data, there is a possibility of recall or recruitment bias in our sample. Interviewed patients differed slightly in terms of race/ethnicity (i.e., interviewed patients were more likely to be African-American than the rest of the cohort and less likely to be Hispanic/Latino). They had similar distributions of psychiatric and substance use diagnoses, although the reported proportions only represent patients receiving clinical services related to these conditions and may not be reliable due to the inaccuracy of ICD-10 coding. To check for possible differential housing instability, we compared the EHR documentation of housing instability between interviewed and non-interviewed patients and found the two groups to be similar in terms of the frequency of documented housing instability, offering some assurance against recruitment bias; however, such bias remains a possibility. Second, we treated each indicator of housing instability as dichotomous. However, accuracy may be improved by factoring information such as the number of notes processed by the NLP during a single encounter or different levels of structured data (e.g., ICD-10 codes indicating risk of homelessness vs. literal homelessness). Third, we examined a cohort of VA patients from one geographic area enrolled in a particular housing program. The observed patterns here of housing instability and EHR documentation may not generalize to other cohorts of Veterans or to populations outside of the VA, who demonstrate different demographic characteristics and documentation patterns. However, documentation of housing and other SDoH is common in clinical texts, and ICD-10 codes are widely used across healthcare systems. We demonstrated here that ReHouSED could be tailored for a new cohort and analysis task, and other work has demonstrated the feasibility of customizing NLP systems developed in VA to be applied in other settings (Chapman et al., 2022). Additionally, this analysis was performed using data from the VA's legacy EHR, VISTA, which is planned to be replaced by Cerner. Future work should compare these findings with data in Cerner to ensure continuing data quality and accuracy.

## 5. Conclusions

Longitudinal housing status is an important outcome for patients who have experienced homelessness. For a sample of 61 homeless-experienced VA patients enrolled in a case management program, we found that housing status was documented longitudinally in the EHR using several structured and unstructured data elements. Using NLP to extract information from clinical notes can improve sensitivity for assessing housing outcomes, while incorporating multiple EHR indicators of housing instability achieves higher specificity compared to single indicators. Future work could customize ReHouSED for processing clinical texts within and outside VA for distinct patient cohorts, augmented by other EHR elements. Similar approaches could also be employed to evaluate other SDoH variables longitudinally using NLP.

## Data availability statement

The datasets presented in this article are not readily available because due to the sensitive and protected nature of this data, the authors are unable to make it available to the public. Requests to access the datasets should be directed to alec.chapman@hsc.utah.edu.

## Ethics statement

The studies involving human participants were reviewed and approved by VA Central Institutional Review Board, U.S. Department of Veterans Affairs. Written informed consent for participation was not required for this study in accordance with the national legislation and the institutional requirements.

## Author contributions

AC contributed to conceptualization, study design, NLP system development, data curation, data analysis, system evaluation, and manuscript preparation. KCo and SG contributed to conceptualization, study design, data curation, system evaluation, and manuscript preparation. SC, TP, and DA contributed to conceptualization, study design, data curation, primary data collection, and manuscript preparation. NJ contributed to conceptualization, study design, data analysis, and manuscript preparation. KCl and JT contributed to data curation and manuscript preparation. RN and AM contributed to conceptualization, study design, NLP system development, data curation, and manuscript preparation. EF contributed to conceptualization, study design, and manuscript preparation. All authors contributed to the article and approved the submitted version.
